# Vascular Complications in Routine Hepatobiliary Procedures and Their Management: An Interventional Radiologist’s Perspective and a Case Series

**DOI:** 10.7759/cureus.99446

**Published:** 2025-12-17

**Authors:** Biswajit Sahoo, Manas Kumar Panigrahi, Pradosh Kumar Sarangi, Nimisha Mishra, Arunprakash Pitchaimuthu, Manoj Kumar Nayak

**Affiliations:** 1 Radiodiagnosis and Interventional Radiology, All India Institute of Medical Sciences, Bhubaneswar, Bhubaneswar, IND; 2 Gastroenterology, All India Institute of Medical Sciences, Bhubaneswar, Bhubaneswar, IND; 3 Radiodiagnosis, All India Institute of Medical Sciences, Deoghar, Deoghar, IND

**Keywords:** complication, endoscopic retrograde cholangiopancreatography, interventional radiology, percutaneous transhepatic biliary drainage, transjugular liver biopsy

## Abstract

Hepatic and biliary interventions, though routinely performed, can lead to significant vascular complications such as bleeding or pseudoaneurysm formation. These injuries usually result from vessel trauma during needle puncture, catheter manipulation, or stent placement and can cause life-threatening hemorrhage if unrecognized. Interventional radiology offers minimally invasive, effective alternatives to surgical management through timely diagnosis and targeted embolization. We present four cases of vascular injuries following common hepatobiliary procedures, each successfully managed using endovascular techniques, underscoring the critical role of interventional radiology in their prompt detection and treatment.

## Introduction

Hepatic and biliary procedures, such as percutaneous biopsy, transjugular liver biopsy (TJLB), percutaneous transhepatic biliary drainage (PTBD), and endoscopic retrograde cholangiopancreatography (ERCP), are essential routine diagnostic and therapeutic tools for a wide range of hepatic, biliary, and pancreatic conditions. Percutaneous and TJLB are indicated for histopathological evaluation of diffuse or focal liver disease; PTBD is used to relieve biliary obstruction or provide biliary access; and ERCP serves both diagnostic and therapeutic purposes in choledocholithiasis, biliary strictures, and pancreatic ductal pathologies. However, these routine procedures come with risks, with vascular injuries and bleeding representing significant complications [1]. These issues often arise from trauma to the vessel wall due to needle punctures or catheter manipulation. If not addressed promptly, they can result in life-threatening hemorrhage. Interventional radiology plays a crucial role in managing these complications, utilizing techniques such as coil embolization to achieve precise and targeted occlusion of the affected vessel, thereby reducing further blood loss, protecting the function of nearby organs, and minimizing the need for high-risk surgeries [2,3]. This case series showcases four distinct instances of vascular complications that arose following routine hepatobiliary procedures, which were effectively managed through interventional radiology.

## Case presentation

Case 1

**Figure 1:**
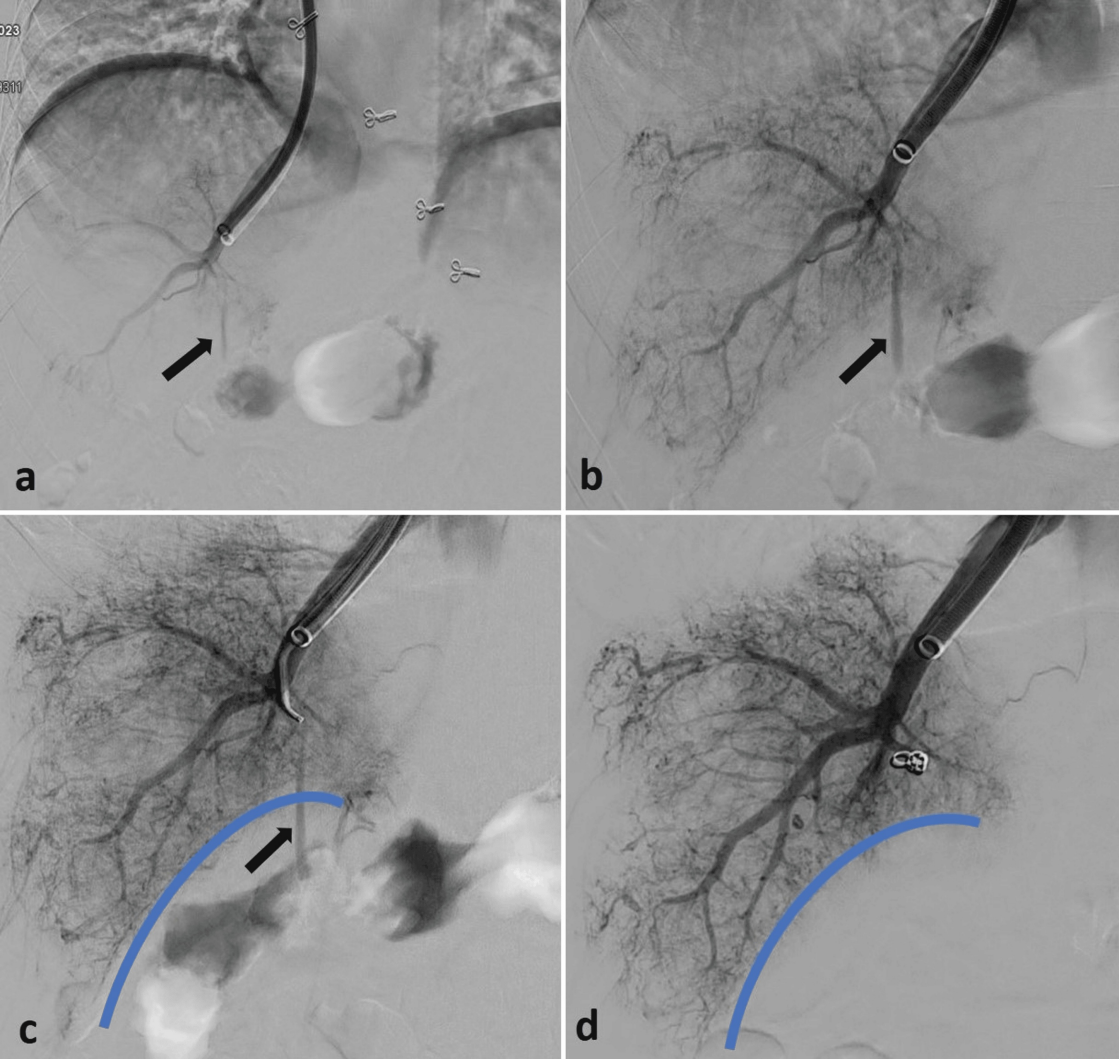
Post-TJLB DSA image (a) shows a faint linear streak of contrast leakage (black arrows) from the biopsy site into the peritoneum, which is better appreciated in the magnified view (b). The biopsy tract was selectively catheterized using a microcatheter (c) and embolized with pushable embolization coils (d). The thick blue line depicts the liver surface. DSA: digital subtraction angiography; TJLB: transjugular liver biopsy

A TJLB was performed on a 45-year-old woman suffering from chronic liver disease of an unknown cause. She exhibited significant ascites and a low platelet count of 24,000/mm3. Following the biopsy, a venogram of the hepatic vein revealed a linear streak of contrast leaking into the peritoneum at the biopsy site (Figure 1a). A subsequent magnified venogram confirmed active bleeding from the biopsy tract (Figure 1b). To address this, the biopsy tract was selectively catheterized with a 5 French Picard catheter (Cook, Bloomington, IN) and a microcatheter (Progreat, Terumo, Japan), after which it was embolized using two small pushable coils (Nester, Cook, Bloomington, IN) (Figures 1c-1d).

Case 2

**Figure 2:**
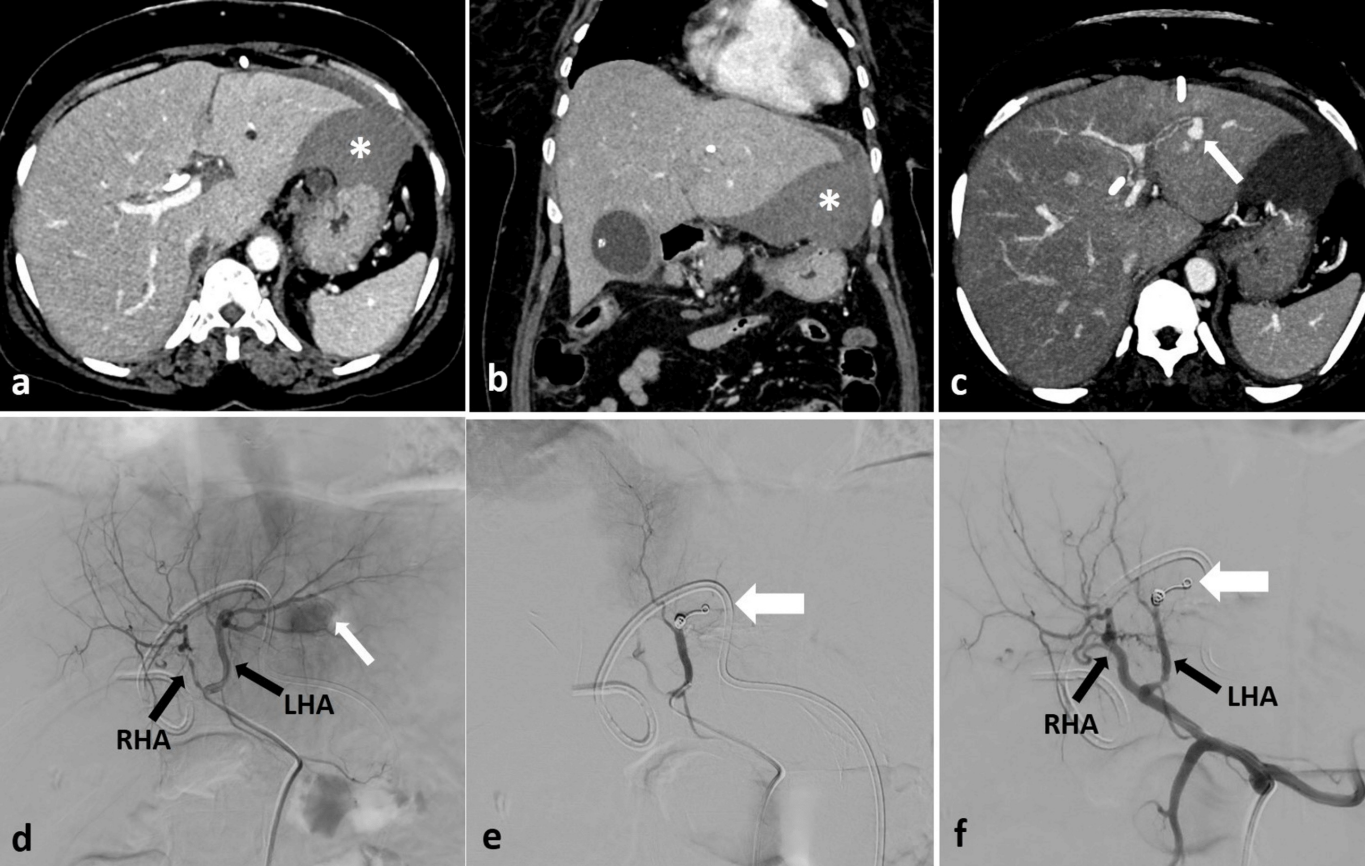
CECT axial (a) and coronal (b) images reveal a significant perihepatic hematoma (asterisk) surrounding the left lobe of the liver after the PTBD. (c) A pseudoaneurysm (thin white arrow) is noted, originating from the left hepatic artery (LHA). DSA images (d-f) illustrate the pseudoaneurysm (thin white arrow) from the LHA, which underwent superselective embolization using pushable coils (thick white arrows). The PTBD tube is visible in situ. Right hepatic artery (RHA). CECT: contrast-enhanced computed tomography; PTBD: percutaneous transhepatic biliary drainage

A 52-year-old female patient with unresectable gallbladder carcinoma presented with a blockage at the common hepatic duct level. To address the obstructive jaundice, PTBD was carried out. The following day, a follow-up blood test revealed a 2-unit drop in the patient's hemoglobin. An ultrasound was conducted, revealing a large perihepatic hematoma around the liver's left lobe. The patient's vital signs remained stable. A contrast-enhanced computed tomography (CECT) scan was performed, showing a pseudoaneurysm from the left hepatic artery along with the perihepatic hematoma (Figures 2a-2c). The patient was then moved to the interventional suite, where a selective hepatic artery angiogram confirmed the CECT findings. The left hepatic artery was selectively catheterized using a microcatheter (Progreat, Terumo, Japan), and the pseudoaneurysm was embolized with coils (Nester) (Figures 2d-2f).

Case 3

**Figure 3:**
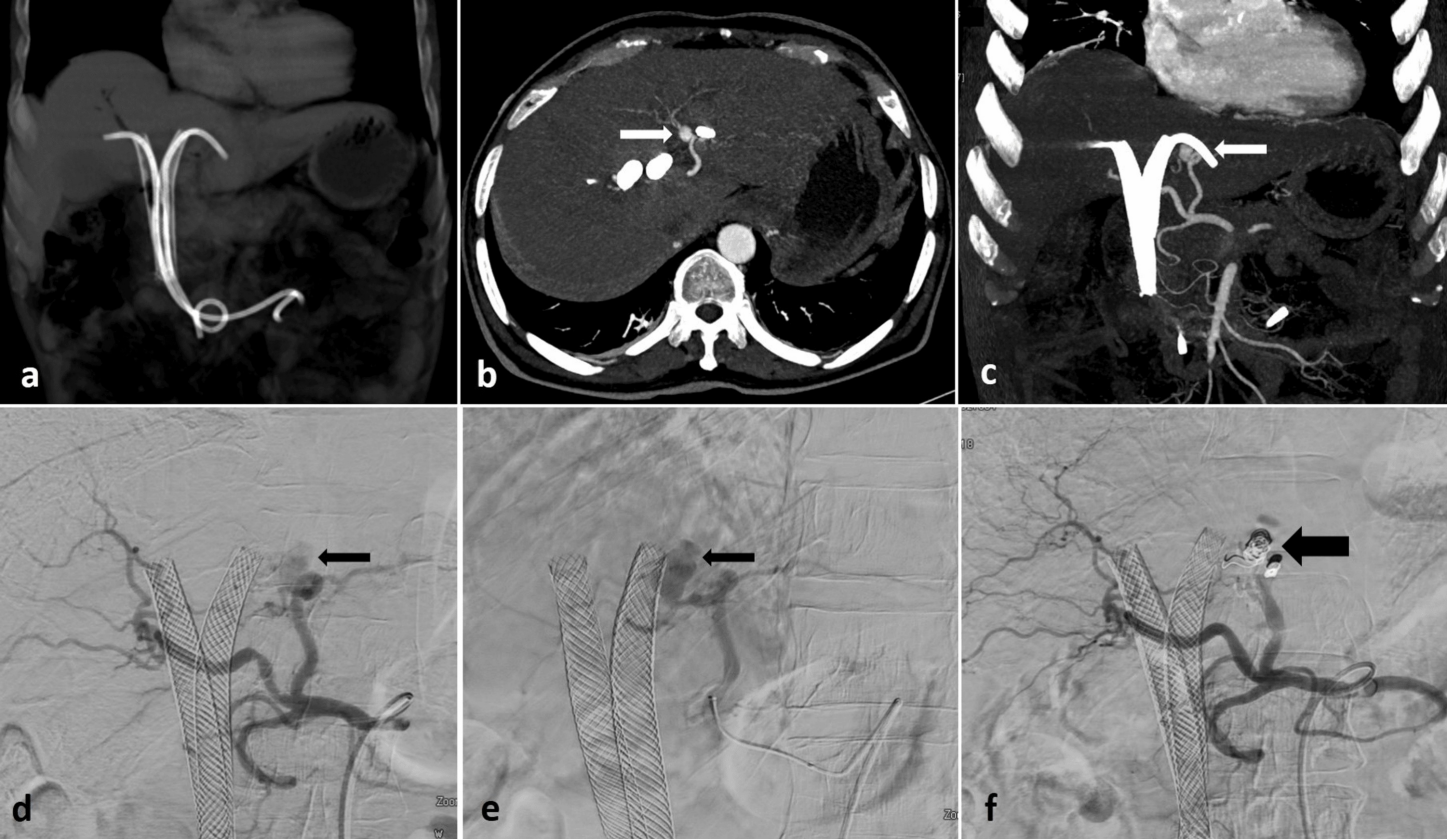
The non-contrast coronal CT image (a) displays two metallic stents with plastic stents positioned across them. CECT axial (b) and coronal (c) arterial phase images reveal a small pseudoaneurysm (indicated by white arrows) forming from the left hepatic artery, closely associated with the left plastic stent. DSA images (d, e) illustrate the pseudoaneurysm from the left hepatic artery (noted by thin black arrows), which was embolized (f) using pushable coils (marked by a thick black arrow). The plastic stents were extracted prior to the embolization procedure. DSA: digital subtraction angiography

A 63-year-old male with unresectable hilar cholangiocarcinoma underwent ERCP for metallic biliary stenting (Epic, Boston Scientific, Marlborough, MA) as a palliative measure. Six weeks after the stent placement, the patient experienced stent block cholangitis and increasing bilirubin levels. Emergency ERCP was performed, deploying plastic stents across the blocked metallic stents. On the 10th day following the plastic stent insertion, he had multiple episodes of upper gastrointestinal bleeding alongside rising bilirubin levels, prompting a CECT scan. This scan revealed a pseudoaneurysm in the left hepatic artery adjacent to the left plastic stent, as well as a hyperdense blood clot inside the metallic stents (Figures 3a-3c). The patient's vital signs remained stable upon presentation. The plastic stents were subsequently removed, and the patient was taken to the interventional suite for pseudoaneurysm management. An angiogram confirmed the CECT findings, revealing the left hepatic artery pseudoaneurysm, which was embolized using pushable coils (Nester) (Figures 3d-3f). Following embolization, two new plastic stents were inserted across the metallic stents, and the patient was discharged after five days without further bleeding episodes.

Case 4

**Figure 4:**
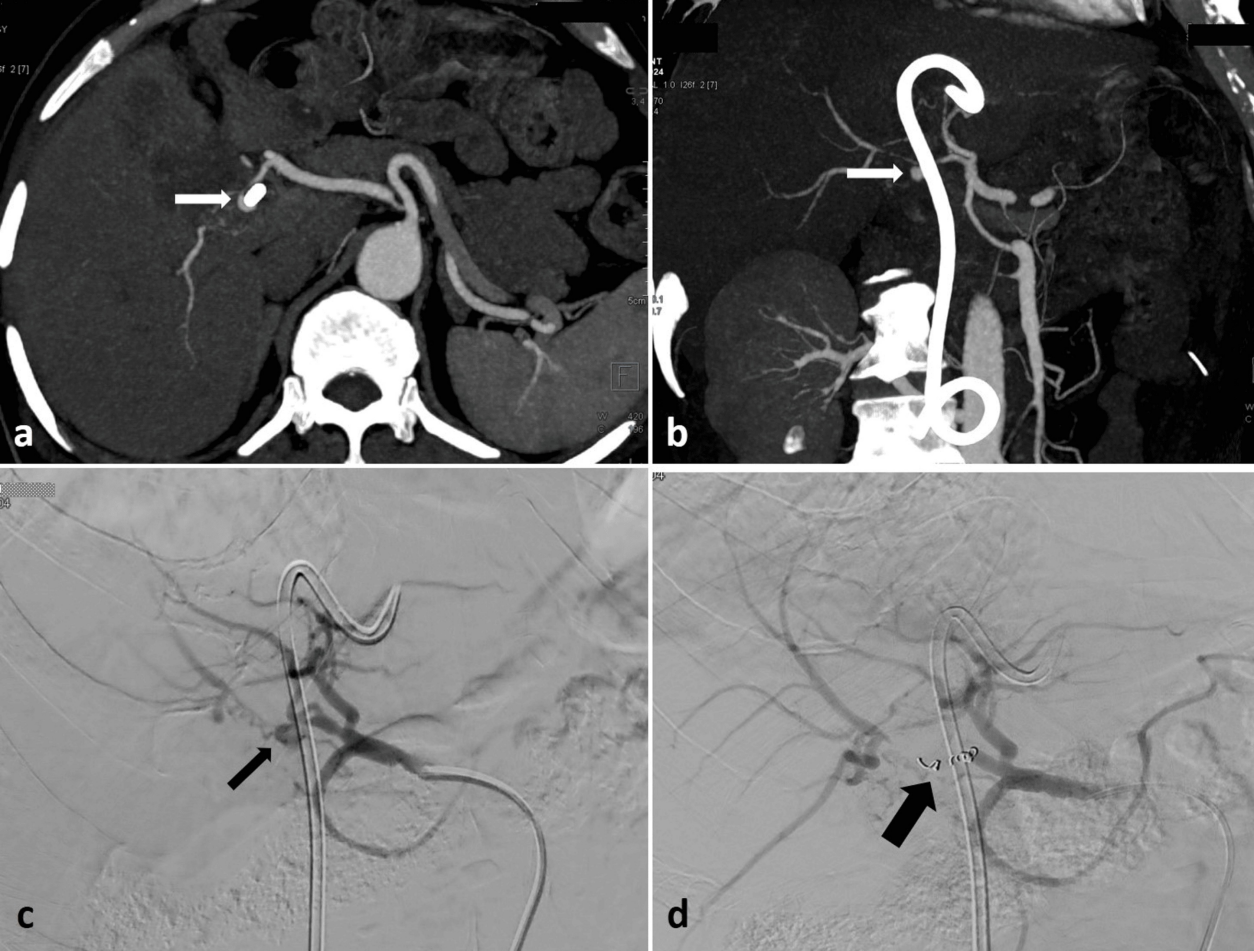
CECT axial (a) and coronal (b) arterial phase images reveal a small pseudoaneurysm (indicated by white arrows) originating from the right hepatic artery near the plastic stent. DSA images (c, d) illustrate the right hepatic artery pseudoaneurysm (shown with a thin black arrow), which was embolized using pushable coils (thick black arrow). CECT: contrast-enhanced computed tomography, DSA: digital subtraction angiography

A comparable situation occurred when a plastic stent, guided by ERCP, was inserted into the biliary system of a 73-year-old man with gallbladder carcinoma, aiming to alleviate his hyperbilirubinemia. On the third day after the stent placement, he experienced severe upper gastrointestinal bleeding. The patient received resuscitation through intravenous fluids, blood transfusions, and inotropes before being transferred for CECT. The CECT revealed a pseudoaneurysm near the plastic stent, originating from the right hepatic artery (RHA) (Figure 4a-4b). He was quickly taken to the interventional suite to address the hepatic artery pseudoaneurysm. The angiogram confirmed the CECT results, showing a pseudoaneurysm in the RHA with active bleeding. Selective catheterization of the RHA was performed using a microcatheter (Progreat, Terumo, Japan), and the pseudoaneurysm was embolized with pushable coils (Nester, Cook) (Figures 4c-4d). The patient was discharged after four days without any further bleeding episodes.

## Discussion

Vascular complications, while uncommon, pose serious risks during hepatobiliary procedures. Such issues can result from direct damage to blood vessels, mishandling during the procedure, or erosion of vessels due to adjacent pathologies or the use of catheters and stents.

Case insights

Transjugular Liver Biopsy (TJLB) (Case 1)

TJLB is a common procedure frequently chosen for patients with coagulopathy (low platelets or prolonged prothrombin time) or ascites, as it lowers the risk of intraperitoneal bleeding when compared to percutaneous biopsy. In contrast to a percutaneous biopsy, a transjugular method lowers bleeding risk by not puncturing the liver capsule, with any biopsy-related bleeding being redirected back into the venous system. Despite its benefits, TJLB does involve certain risks, categorized into minor complications, which occur at a rate of 6.5%, including neck hematoma and carotid puncture. Major complications occur at a rate of 0.6% and may involve conditions such as intraperitoneal hemorrhage from an extracapsular liver puncture, hepatic hematoma, hepatic artery pseudoaneurysm, hemobilia, cardiac arrhythmia, and pneumothorax [4]. Liver capsule breach, though uncommon, can occur, particularly in a small contracted liver. To detect potential complications early, such as hepatic artery injury or capsular breach, a thorough post-biopsy venogram of the hepatic vein must be performed as standard practice. However, in some instances, it may be impossible to identify such complications through a post-biopsy hepatic vein venogram; thus, an emergent CECT can be conducted when there is a high clinical suspicion of complications. If a capsule breach occurs, the biopsy tract should be embolized immediately using coils or n-butyl-2-cyanoacrylate (NBCA). Selective catheterization and coil embolization have proven effective in controlling bleeding, highlighting the significance of post-biopsy imaging in detecting complications [5].

Percutaneous Transhepatic Biliary Drainage (PTBD) (Case 2)

PTBD is an essential routine procedure for addressing obstructive jaundice, particularly in cases involving unresectable tumors. However, unintentional vessel damage during needle insertion or catheter handling can lead to hemobilia, pseudoaneurysm formation, or injury to the portal vein. An emergent ultrasound and CECT should be performed. If the bleeding site involves a peripheral branch of the hepatic artery or portal vein, embolization can be done using coils or NBCA. Other alternative techniques, such as balloon tamponade or the placement of a covered stent, can also be considered if the bleeding originates from the first and second-order segments of the hepatic artery or portal vein. It is essential to manage the bleeding site, whether it is the portal vein or hepatic artery, to stabilize the patient, highlighting the importance of careful imaging guidance throughout the PTBD process and thorough monitoring following the procedure [6]. 

Endoscopic Retrograde Cholangiopancreatography (ERCP) (Cases 3 and 4)

ERCP may result in various complications, including vascular injuries caused by stent placement. Pseudoaneurysm formation or active bleeding can arise from mechanical trauma or erosion of adjacent vessels by stents (plastic or metallic). Rapidly pinpointing the source of bleeding through CECT or DSA, followed by coil or NBCA embolization or placement of a covered stent, is essential for survival [7].

The selection among coil embolization, NBCA embolization, and the placement of a covered stent is heavily contingent upon specific characteristics such as aneurysm size and location, vessel tortuosity, blood flow dynamics, and the necessity of preserving the parent artery. Coil embolization may be employed in cases involving small aneurysms, aneurysms with narrow necks, or within small, tortuous vessels where the preservation of parent artery flow is of lesser importance. In contrast, stenting is generally preferred for large, straight arteries, vessel perforations, or wide-necked aneurysms, where maintaining blood flow in the parent artery is paramount. Complex cases, where standard embolization methods (coils or covered stents) are unfeasible due to vessel tortuosity, small diameter, or the exigency of rapid, complete sealing of a bleeding site, typically necessitate NBCA embolization.

Interventional radiology is vital in managing vascular issues within the hepatobiliary system, offering several advantages. First, embolization selectively occludes the bleeding vessel while maintaining blood circulation to surrounding tissues. Second, this minimally invasive approach reduces procedural risks and accelerates recovery compared to conventional surgery. Finally, interventional radiologists collaborate closely with gastroenterologists, surgeons, and intensivists to improve patient outcomes [8]. In cases of suspected bleeding following percutaneous biopsy, PTBD, or ERCP-guided interventions, imaging methods such as ultrasound, CECT, and DSA are crucial for identifying the source of bleeding and informing further intervention strategies. For TJLB, the most critical step is performing a post-biopsy venogram to assess for an accidental capsular breach, a hepatic artery pseudoaneurysm, or hemobilia. Occasionally, CECT may be necessary to uncover occult bleeding in these cases. Treatment options depend on the patient's hemodynamic status. Surgical intervention is required in unstable patients, non-localization of the source of bleeding, or after failed angiographic embolization. Most of the patients are hemodynamically stable, and interventional radiological procedures are the initial treatment modality. Factors such as coagulopathy, ascites, and malignancy-related vascular fragility increase the risk of complications, influencing the choice of embolization materials and techniques. Pushable embolization coils provide effective hemostasis while ensuring procedural convenience. However, in more complex cases, alternative embolic agents, like liquid embolics (NBCA) and vascular plugs, may be essential [9,10]. In some scenarios, a covered stent can also be placed.

## Conclusions

Although the incidence of severe hemorrhage following routinely performed hepatobiliary procedures is very low, it is important to remember that every procedure carries risks. Interventional radiology plays a vital role in addressing these life-threatening issues through minimally invasive techniques. Our four cases demonstrate some of the hemorrhagic complications that can arise after routine hepatobiliary procedures and can be treated by an interventional approach. Timely diagnosis, a collaborative approach, and proficient use of embolization techniques are crucial for achieving successful results.
